# Delayed Cerebral Ischemia After Subarachnoid Hemorrhage: Is There a Relevant Experimental Model? A Systematic Review of Preclinical Literature

**DOI:** 10.3389/fcvm.2021.752769

**Published:** 2021-11-15

**Authors:** Suzanne Goursaud, Sara Martinez de Lizarrondo, François Grolleau, Audrey Chagnot, Véronique Agin, Eric Maubert, Maxime Gauberti, Denis Vivien, Carine Ali, Clément Gakuba

**Affiliations:** ^1^CHU de Caen Normandie, Service de Réanimation Médicale, Caen, France; ^2^Normandie University, UNICAEN, INSERM, U1237, PhIND ≪ Physiopathology and Imaging of Neurological Disorders ≫, Institut Blood and Brain @ Caen-Normandie, Cyceron, Caen, France; ^3^Centre d'Epidémiologie Clinique, AP-HP (Assistance Publique des Hôpitaux de Paris), Hôpital Hôtel Dieu, Paris, France; ^4^CHU Caen, Department of Clinical Research, CHU Caen Côte de Nacre, Caen, France; ^5^CHU de Caen Normandie, Service d'Anesthésie-Réanimation Chirurgicale, Caen, France

**Keywords:** delayed cerebral ischemia, experimental models, subarachnoid hemorrhage, vasospasm, systematic review

## Abstract

Delayed cerebral ischemia (DCI) is one of the main prognosis factors for disability after aneurysmal subarachnoid hemorrhage (SAH). The lack of a consensual definition for DCI had limited investigation and care in human until 2010, when a multidisciplinary research expert group proposed to define DCI as the occurrence of cerebral infarction (identified on imaging or histology) associated with clinical deterioration. We performed a systematic review to assess whether preclinical models of SAH meet this definition, focusing on the combination of noninvasive imaging and neurological deficits. To this aim, we searched in PUBMED database and included all rodent SAH models that considered cerebral ischemia and/or neurological outcome and/or vasospasm. Seventy-eight publications were included. Eight different methods were performed to induce SAH, with blood injection in the *cisterna magna* being the most widely used (*n* = 39, 50%). Vasospasm was the most investigated SAH-related complication (*n* = 52, 67%) compared to cerebral ischemia (*n* = 30, 38%), which was never investigated with imaging. Neurological deficits were also explored (*n* = 19, 24%). This systematic review shows that no preclinical SAH model meets the 2010 clinical definition of DCI, highlighting the inconsistencies between preclinical and clinical standards. In order to enhance research and favor translation to humans, pertinent SAH animal models reproducing DCI are urgently needed.

## Introduction

**Figure 1:**
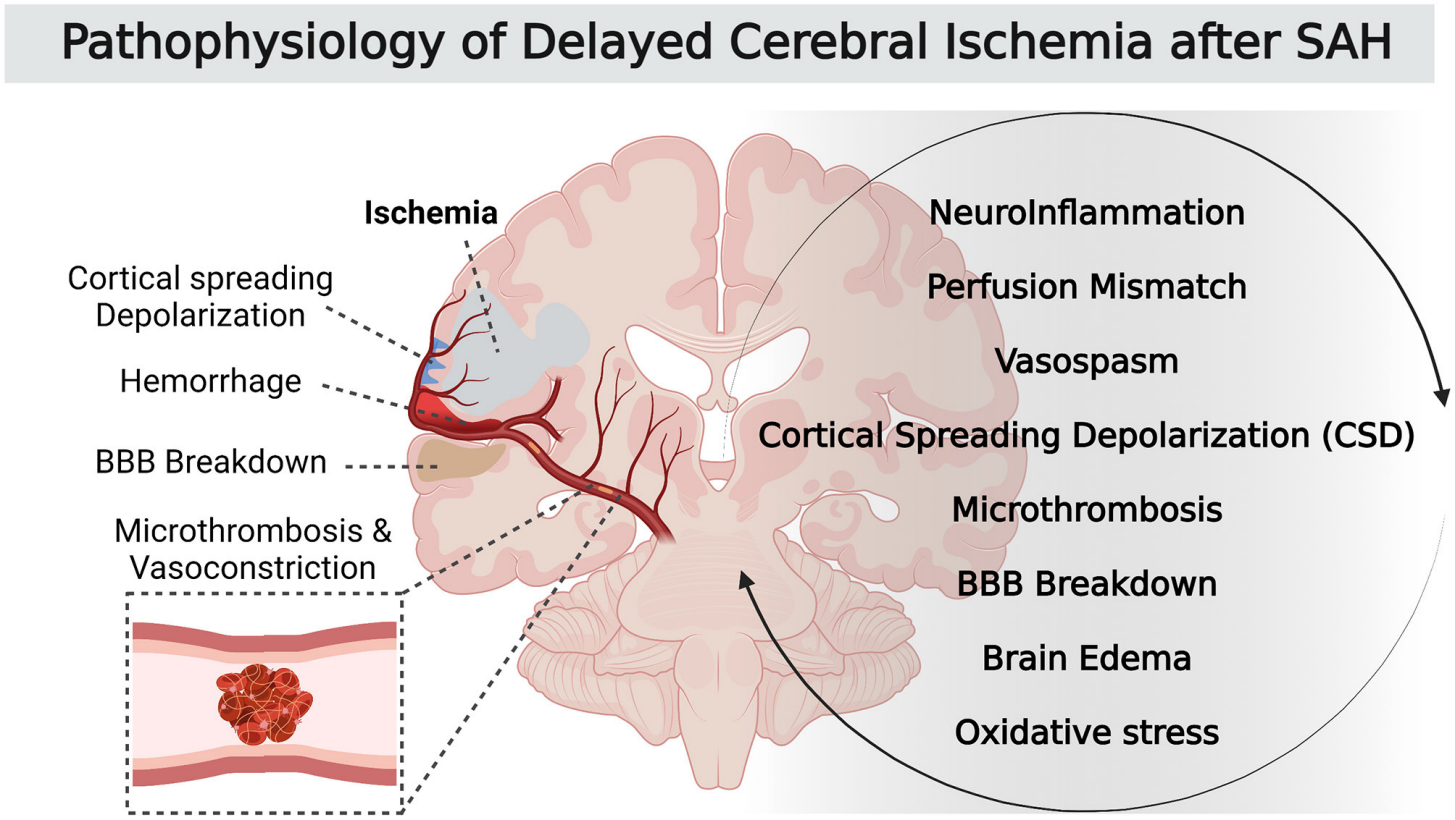
Pathophysiology of DCI after SAH.

Subarachnoid hemorrhage (SAH) is a neurological emergency characterized by the extravasation of blood into subarachnoid spaces. Around 80% of non-traumatic subarachnoid hemorrhage result from the rupture of an intracranial aneurysm, and have a high rate of death and complications. Aneurysmal SAH is therefore one of the most frequent causes of admission in neurocritical care. Delayed cerebral ischemia (DCI) occurs in ~30% of cases after aneurysmal SAH (1) and is the leading cause of morbidity for surviving SAH patients. To date, no treatment of DCI improves neurological outcome. Unfortunately, the exact mechanisms of DCI pathophysiology remain poorly understood. The current consensus suggests that the origin of DCI is a multifactorial and complex process. It not only includes the narrowing of cerebral arteries (i.e., vasospasm) but also the activation of others pathways, including a neuroinflammatory reaction that promotes perfusion mismatch with neurovascular uncoupling, as well as other pathological phenomena such as microthrombosis, cortical spreading depolarization and breakdown of the blood-brain barrier (Figure 1) (2, 3). All these local and systemic inflammatory responses are involved in the genesis and development of DCI.

These different mechanisms start at ictus, during early brain injury, and result in neuronal injury and sometimes in parenchymal infarction. Large vessel vasospasm was commonly recognized as the main factor leading to DCI after SAH. In fact, recent studies support that large vessel narrowing is a delayed contributor to a cascade of events that starts earlier during the acute phase after SAH. This critical earlier phase with multifactorial pathophysiological pathways is probably the most promising therapeutic target to improve patient outcomes. To better understand SAH and its complications and to facilitate the development of an effective treatment, many animal models have been developed. The lack of a consensual definition of DCI led to a large diversity of terms used and parameters studied. As a result, findings from preclinical research were controversial (4). But in 2010, an international expert panel involved in SAH research developed a definition of DCI in humans. The consortium decided that a uniform definition of DCI should capture both cerebral infarction (imaging) and clinical deterioration (functional) elements in terms of morphological and clinical characteristics. They stated that in clinical trials aiming to develop therapeutics against DCI after SAH, the two main outcome measures should be: (1) infarction identified on computed tomography (CT) or magnetic resonance imaging (MRI) or proven at autopsy, after exclusion of procedure-related infarctions and (2) functional outcome (3).

The definition of DCI-related cerebral infarction was as follows: diagnosis of cerebral infarction performed by either a brain CT or MR scan within 6 weeks after SAH, or on the latest CT or MRI scan made before death within 6 weeks, or proven at autopsy, not present on the CT or MRI scan between 24 and 48 h after early aneurysm occlusion, and not attributable to other causes, such as surgical clipping or endovascular treatment.

Regarding the functional outcome, experts specified that the definition of clinical deterioration caused by DCI is the occurrence of focal neurological impairment (such as hemiparesis, aphasia, apraxia, hemianopia, or neglect), or a decrease of at least 2 points on the Glasgow Coma Scale, which is not apparent immediately after aneurysm occlusion, and cannot be attributed to other causes.

Based on clinical assessment in humans and considering the occurrence of DCI as the main determinant for the functional outcome, this consensus-building approach allowed to determine that the ideal SAH model should associate both the occurrence of cerebral infarction evidenced on brain imaging or histology and some altered functional outcome. In a translational perspective, noninvasive imaging is preferable to histology for several reasons. Brain imaging is the most common way to investigate cerebral ischemia in clinical trials, and ischemia proved on brain imaging is up to date the only paraclinical outcome that was improved with use of therapeutics that enhance SAH-patients functional outcome (5). Moreover, infarction is well defined by imaging in contrast to histology, which is difficult to define with a consensual diagnostic method.

This systematic review aimed at identifying and analyze the different murine models of SAH, and to describe the extent to which they meet the human definition of DCI, i.e., more specifically according to the associating of the two most relevant evaluation criteria that are the proof of brain ischemia with imaging and the occurrence of neurological deficits.

## Materials and Methods

### Systematic Search

This systematic review was reported following the Preferred Reporting Items for Systematic Reviews and Meta-analysis (PRISMA) statement (6, 7). We searched the PUBMED database on July 1, 2020 with the search terms “subarachnoid hemorrhage,” “models, animal,” “mice,” “rats,” “vasospasm, intracranial,” and “delayed cerebral ischemia.” Abstracts from relevant congresses were also considered. Two authors independently screened the titles and abstracts and reviewed the full text of any potentially eligible publication. Divergences were resolved by consensus.

### Eligibility Criteria for Included Animal Studies

Studies were included if they involved (1) description and/or modification of a subarachnoid hemorrhage model in rats or mice (2) study of arterial cerebral vasospasm and/or ischemia and/or neurological outcome. Systematic reviews and meta-analyses as well as *in vitro* studies were excluded. Among the experimental studies developing a method and/or assessing therapeutic strategies, only those, which described a new SAH model, were included The included studies were limited to articles written in English, Spanish, German, Russian, Italian, Portuguese, and French. There was no restriction for year of publication.

### Data Collection

For each study, we extracted the journal and authors names, the year of publication, number of citations for each article and the impact factor corresponding to that year. Two publications were classified as coming from the same team if they had one or more author in common considering only authors in first, second, last or penultimate position. Animal characteristics were extracted as follows: species, strain, sex, and weight; model of SAH as follows: method of induction, vascular territory, rupture of an aneurysmal vessel, location of blood injection, using of a pharmaceutical adjuvant to induce ischemia, characteristics of blood used (nature, volume, and number of injections); anesthesia and monitoring as follows: general anesthesia, mechanical ventilation, temperature, blood glucose levels, cerebral blood flow, intracranial pressure, and blood pressure monitoring; study of vasospasm as follows: method (imaging, histology, times studies of vasospasm, study of cerebral blood flow); study of cerebral ischemia as follows: method (imaging, histology, times studies of ischemia, topography, and related searches like neuroinflammation, microthrombosis or microglial activation); mortality and behavioral study as follows: general condition, weight, sensory-motor, and cognitive tests.

### Statistical Analysis

We represented the median and extreme values (median [minimum—maximum]) of continuous variables, and the number of occurrences with proportions represented as percentages for categorical variables.

## Results

### Included Rodent Studies

**Figure 2:**
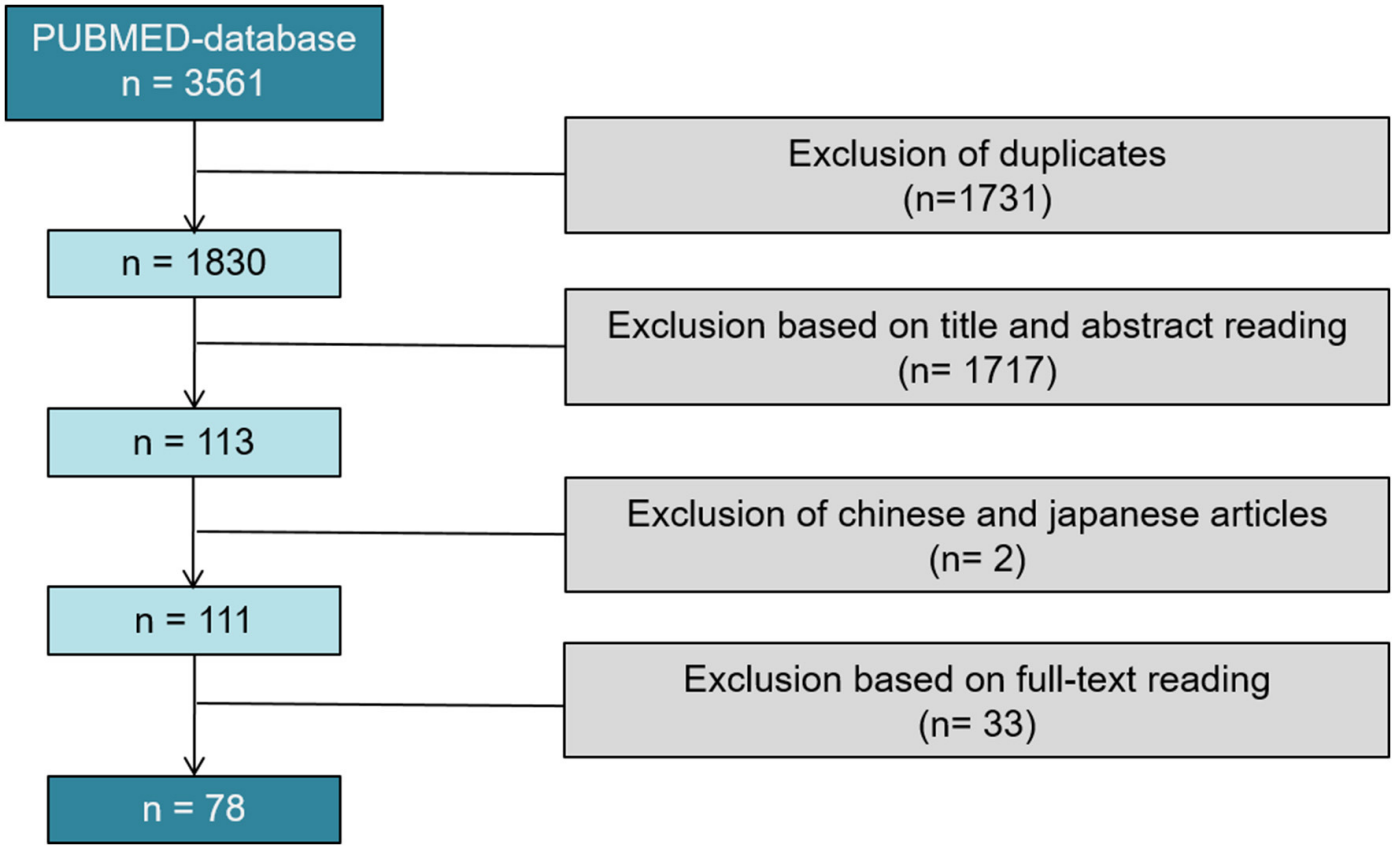
PRISMA flow diagram of the systematic review; 78 studies were included in our systematical review.

Of 3,561 articles, only 78 reports proved eligible (8–85) (Figure 2).

### Characteristics of the Rodent Studies

**Figure 3:**
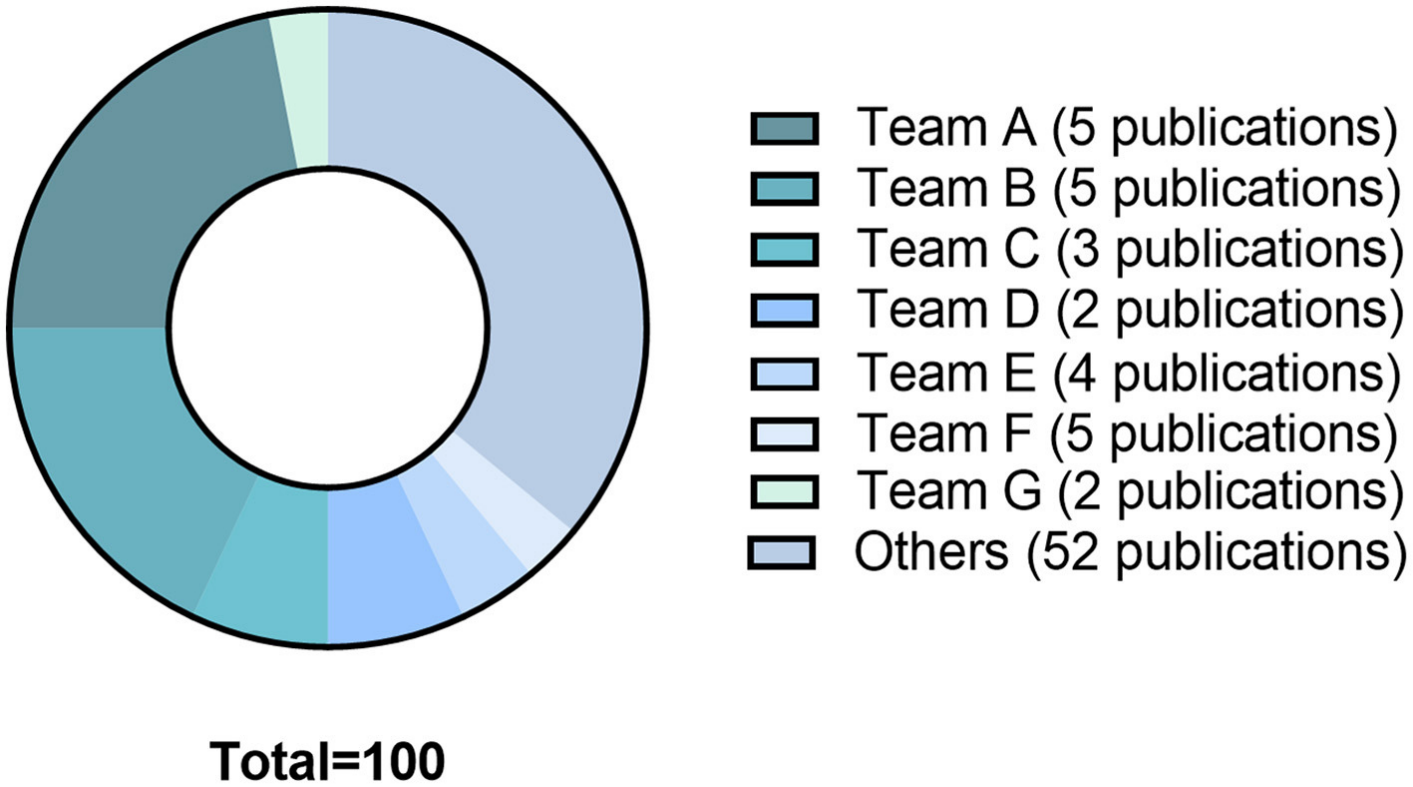
The 78 studies included in the systematical review generated since their publication 3681 citations. The graph shows the distribution of citations by teams: Team A: Bederson, Mount Sinai School of Medicine, New York; Team B: Prunell, Department of Clinical Neuroscience, Section for Neurosurgery, Karolinska Institute, Stockholm, Sweden; Team C: Solomon, Department of Neurological Surgery, Columbia University College of Physicians and Surgeons, New York; Team D: Yamamoto, Department of Neurosurgery, Hamamatsu University School of Medicine, Hamamatsu, Japan; Team E: Macdonald RL, Division of Neurosurgery, University of Alberta, Edmonton, Canada. Team F: Thal Institute for Surgical Research, University of Munich Medical Center—Grosshadern, Munich, Germany; Team G: Warner DS, Department of Anesthesiology, Duke University Medical Center, Durham, North Carolina 27710, USA.

The 78 articles were published in 26 different journals. The median impact factor of the year of publication was 2.45 [0.82-6.12]. The year of publication ranged from 1979 to 2020. Fifty-nine studies (76%) were published between 2,000 and 2020, 27 of which were published following the 2010 article that defined DCI (3). Fifty-three different teams were identified. Half of publications (*n* = 39; 50%) resulted from 14 teams. Five (6%) publications were extracted from team A (Bederson, Mount Sinai School of Medecine, New York), 5 (6%) publications were extracted from team B (Prunell, Department of Clinical Neuroscience, Section for neurosurgery, Karolinska Institute, Stockholm, Sweden), and 3 (4%) publications were extracted from team C (Solomon, the Department of Neurological Surgery, Columbia University College of Physicians and Surgeons, New York). Almost two thirds (64%) of citations were issued from 26 studies published by 7 teams (Figure 3).

### Characteristics of SAH Models in Rats and Mice

**Figure 4:**
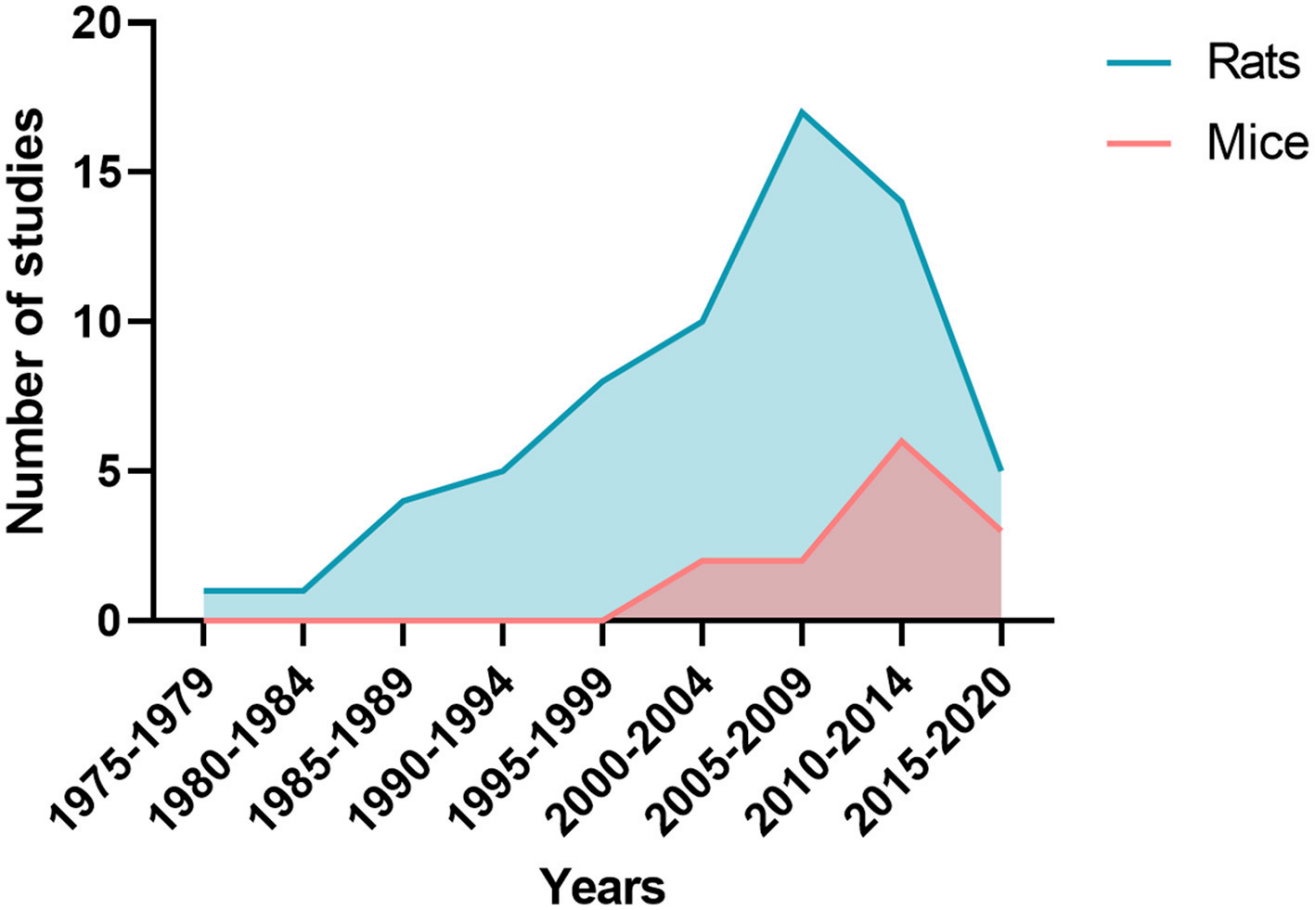
Number of publications by five-year periods depending whether studies were performed on rats or mice.

**Figure 5:**
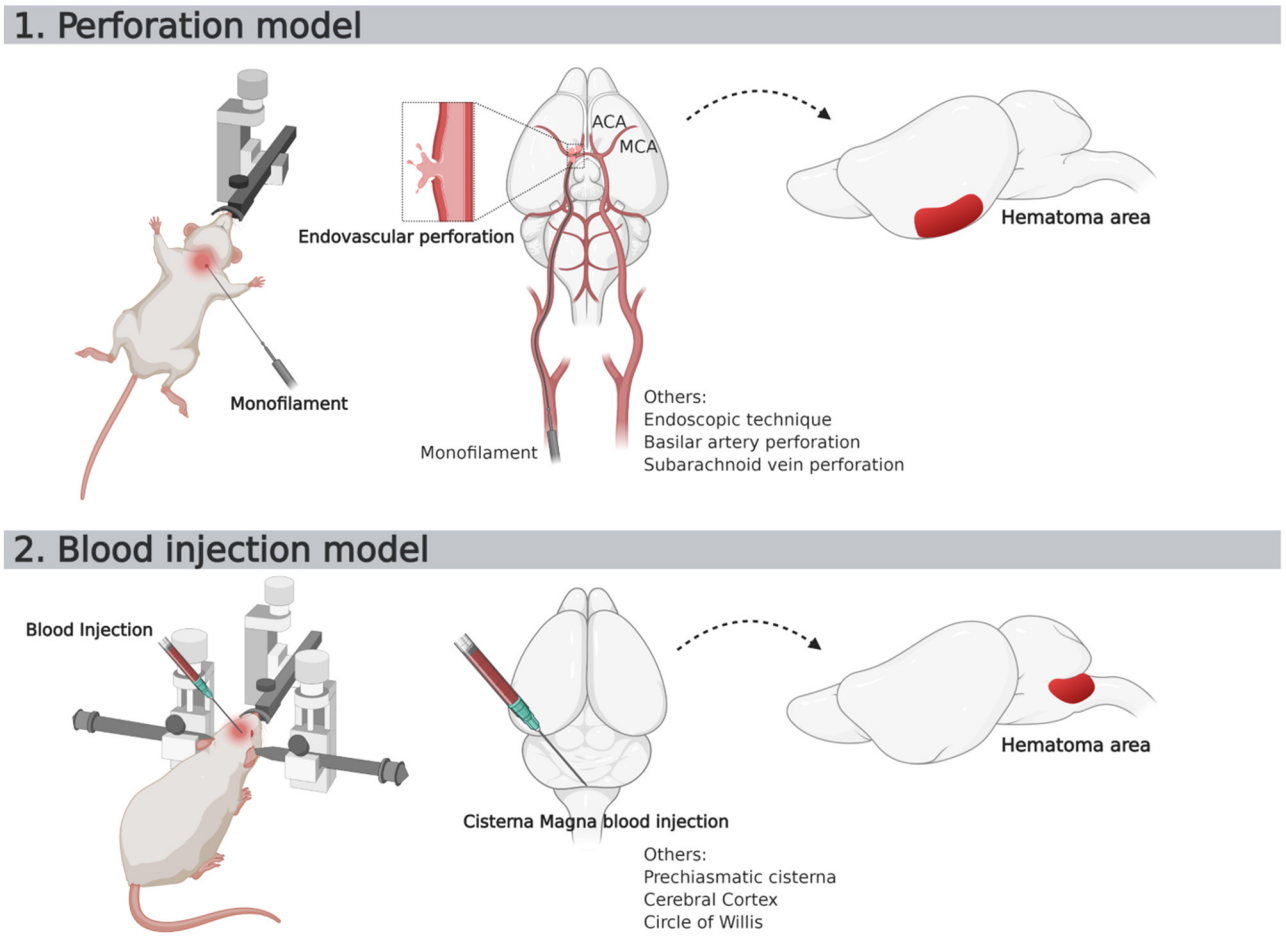
Schematic representation of two most used SAH murine models. ACA, Anterior Cerebral Artery; MCA, Middle Cerebral Artery.

The characteristics of SAH models are summarized in Table 1. The most commonly used species was rats (66 publications, Figure 4). The two main strains of rats were Sprague-Dawley (*n* = 49; 74%) and Wistar (*n* = 17; 26%). Two publications used a model with comorbidity that was diabetes (64) or hypertension (12). No model used female animals. With respect to the surgical procedure, 58% of the models (*n* = 45) corresponded to SAH involving posterior cerebral circulation. The two most frequently used models were blood injection in the *cisterna magna* (*n* = 39; 50%) and endovascular perforation (*n* = 23; 29%) (Figure 5). Among vascular perforation models, four variants were described: endovascular perforation, endoscopic technique, perforation of the basilar artery and perforation of subarachnoid veins. Among the direct injection models, blood was injected into the *cisterna magna*, the pre-chiasmatic cistern, the cerebral cortex or directly into the circle of Willis. Blood was from autologous origin in 97% of publications (*n* = 76). Blood could be arterial (*n* = 67; 92%) or venous (*n* = 7; 10%). The nature of the blood was not specified in 3 studies. The injected blood volume varied across studies: the median blood volume injected was 300 μL [100–700 μL] and 80 μL [50–100 μL] in rats and mice, respectively. Thirty-six studies (47%) described a model with a single injection. Two injections separated by a free period of 24 to 48 h were performed in 15 studies (20%). The aim of these double injection models was to increase the severity of the SAH, while maintaining an acceptable mortality rate. These double injection models were only performed in rats. One model used the induction of hypertension associated with elastase injection into the cerebrospinal fluid in order to promote the aneurysmal rupture (70). Some studies reported the use of adjuvants to promote the occurrence of ischemia. The first study published in 2011 described a direct injection model in insulin-resistant rats (64). Other studies combined induction of SAH by simple or double direct blood injections with the occlusion of the common carotid artery as an ischemia promoting factor (71). One of these models promoted occurrence of DCI, which was associated to the injection of blood, the occlusion of common carotid and the induction of spreading depolarization. Then, authors investigated the effect of an administration of a pro-inflammatory agent, before SAH induction (73).

**Table 1:** Characteristics of SAH models.

**Characteristics of subarachnoid hemorrhage models**	**Number (%)**
*Species*	
Rats	66 (85)
Mice	13 (17)
*Method of induction of SAH*	
Vascular perforation	
Circle of Willis	23 (29)
Basilar artery	4 (5)
Subarachnoid vein	1 (1)
Direct injection	
*Cisterna magna*	39 (50)
Prechiasmatic cistern	12 (15)
Cerebral cortex	1 (1)
Circle of Willis	1 (1)
Induced hypertension and elastase	1 (1)
Models with factors promoting cerebral ischemia	4 (5)
*Number of direct blood injection in the subarachnoid space*	
0	28 (37)
1	36 (47)
2	15 (20)
*Vascular territory of SAH*	
Anterior	38 (49)
Posterior	45 (58)
*Blood*	
Arterial	67 (92)
Venous	7 (10)
No specified	4 (5)
*Management of anesthesia*	
General anesthesia	77 (99)
No specified	1 (1)
Mechanical ventilation	38 (49)
*Monitoring*	
Invasive blood pressure	45 (58)
Intracranial pressure	30 (38)
Global cerebral blood flow	23 (29)
Local cerebral blood flow	21 (27)
Temperature	58 (75)
Glucose level	9 (12)
*Study of cerebral ischemia*	30 (38)
Positive diagnostic of ischemia	24 (26)
Ischemia at distance of subarachnoid hemorrhage	21 (27)
Cerebral cortex	16 (21)
Hippocampus	16 (21)
Cerebellum	1 (1)
Basal ganglia	4 (5)
Diagnostic of cerebral ischemia	
Imaging	3 (4)
Histology	24 (31)
Fluorojade B	5 (6)
Apoptosis	7 (9)
Quantitative assessment	8 (10)
Qualitative assessment	7 (9)
Parameters associated with ischemia	
Microthrombosis	6 (8)
Microglial activation	2 (3)
Inflammation (neutrophil polynuclear labeling)	1 (1)
*Study of vasospasm* Imaging Histology Hematoxylin and eosin Positive diagnostic of vasospasm	51 (67) 25 (32) 34 (44) 20 (26) 48 (62)
*Study of cerebral blood flow* Doppler Magnetic resonance imaging Angiography Others	39 (50) 25 (32) 11 (14) 9 (10) 7 (9)
*Physical and behavioral examination* Neurological assessment Sensory-motor tests Cognitive tests General condition Weight Death	19 (24) 18 (23) 7 (9) 32 (41) 12 (15) 45 (58)

In all studies, the procedure took place under general anesthesia. In 40 studies, the animals were kept in spontaneous ventilation during general anesthesia. An invasive blood pressure monitoring was used in 58% of the studies. Three studies performed an invasive monitoring of blood pressure in mice. Intracranial pressure was monitored during the procedure in 38% of the studies. One study performed an intracranial pressure monitoring in mice with the help of a sensor placed in the *cisterna magna* (42). Blood glucose monitoring was performed in 12% of studies (*n* = 9) and temperature monitoring was carried out in 75% of the studies (*n* = 58).

### Study of Vasospasm

**Figure 6:**
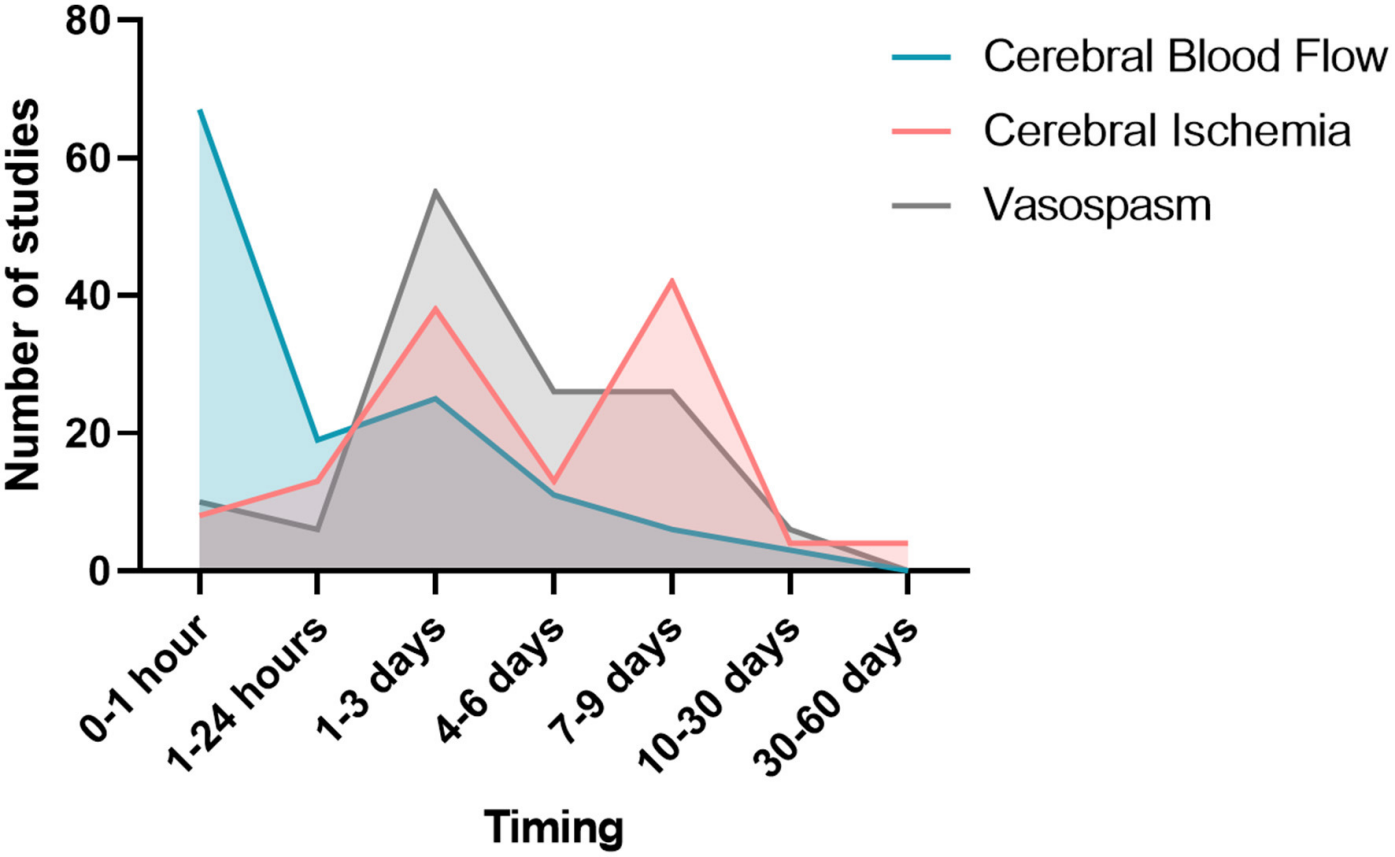
Study window of vasospasm, cerebral blood flow, and cerebral ischemia.

From the 39 studies evaluating cerebral hemodynamics, 37 observed a decreased cerebral blood flow following SAH. The study window of cerebral blood flow varied according to the publications (Figure 6). Twenty-five studies searched the occurrence of arterial vasoconstriction with MRI, Doppler, angiography, videomicroscopy, positron emission tomography (PET) or photomicrography. All of them observed vasospasm. Thirty-four studies assessed vasospasm with histology (Table 1). The main staining was Hematoxylin and Eosin (20 studies). Histological study were performed at different times. The authors diagnosed vasospasm in 30 studies with histology. Vasospasm was studied in a total of 588 animals in 34 publications. The diagnosis was made in 513 animals (87%) but some authors did not specify the number of animals studied.

### Study of Ischemia

Overall, 532 animals were screened for cerebral ischemia in 30 publications. A positive diagnosis was made in 196 animals (37%) but some of these publications (*n* = 9) did not specify the exact number of animals for which the positive diagnosis of ischemia was made. Out of 29 publications searching for ischemia with histological study, 24 reported evidence of ischemia. All these authors described occurrence of ischemia remotely from the origin of SAH. The existence of ischemia was mainly studied in two brain regions: the cortex and the hippocampus. The other brain regions explored were the cerebellum and basal ganglia. Different methods for studying ischemia were used in histology. Neuronal quantification was the main method (in 8 studies). The detection of apoptosis appeared to be the second way to explore ischemia (7 publications). Apoptosis was assessed by TUNEL staining or caspase activity assays. A specific labeling of neuronal death was also used by fluorojade B in 5 studies. Qualitative assessment of neurons was performed in 7 studies. Histological studies of ischemia were achieved at early and/or late times, as summarized on Figure 6. Some publications focused on the pathophysiological mechanisms underlying ischemia. Indeed, several studies have attempted to highlight phenomena of microthrombosis and neuroinflammation. Microthrombosis was evaluated by the presence of fibrin [anti-fibrin(ogen)] and platelet (anti-platelet GpIIbIIIa) aggregates by immunofluorescences studies. Neuroinflammation was evidenced at the local level by histological studies (neutrophils labeling), but also at the systemic level using markers, such as TNF and IL-1β. Two publications studied microglial activation *via* specific immuno-staining of microglia such as Iba-1 (73, 78). Only 4 studies investigated ischemia with MRI.

### Behavioral Evaluation and Body Weight Monitoring

Behavioral assessment was performed in 32 studies. The general status of animals was described using the spontaneous locomotor activity, circling behavior, whisker movements, and coat state. Eighteen studies used sensorimotor tests for the quantitative behavioral assessment. These studies were performed with delays ranging from <24-h to 4 weeks after SAH induction. Three studies used the rotarod test. Tests assessing the cognitive abilities of animals were conducted in 7 studies. Among these studies, 4 used the Morris water maze to assess working memory. Body weight monitoring was carried out in 12 studies.

### Mortality Rate

The mortality rate was reported in 58% of the studies (*n* = 45). However, the mortality study period and the death rate varied between studies. The direct-injection model into the *cisterna magna* was responsible for a mortality rate between 0 and 52%. The prechiasmatic cistern injection pattern induced a mortality rate ranging from 0 to 100%. The endovascular perforation model was responsible for a mortality rate between 6 to 65%.

### Included Non-primates Studies

Finally, we also reviewed non-human primate models keeping the same eligibility criteria. Of 175 articles, citations from 22 reports were proved eligible (86–107). Then, we compared their principal characteristic to the rodent models, as shown on Table 2.

**Table 2:** Comparison between primates and rodents SAH models focusing on main characteristics.

**Characteristics of experimental SAH models**	**Number of publications (%)**	
*Species*	Primates (*n* = 22)	Rodents (*n* = 78)
*SAH induction method*		
Direct blood injection	22 (100)	53 (68)
Vascular perforation	0 (0)	28 (36)
*Study of cerebral ischemia* Imaging Histology	3 (14) 0 (0) 3 (14)	30 (38) 3 (4) 24 (31)
*Study of cerebral vasospasm* Angiography Histology *Positive diagnosis of vasospasm*	21 (95) 20 5 (23) 22 (100)	51 (67) 9 (10) 34 (44) 48 (62)
*Study of cerebral blood flow*	6 (27)	39 (50)
*Neurological examination* Delayed neurological deficits	15 (68) 3 (14)	19 (24) ND

*ND, not documented*.

## Discussion

In this systematic review, we evaluated 78 publications and found that 8 different methods were used to induce hemorrhage in the subarachnoid spaces. These methods can be classified into three groups. In the first group, SAH is due to perforation of an arterial or venular vessel. In the second, a blood injection is performed in cerebrospinal fluid or directly in the brain parenchyma. In the third, SAH is induced by the combination of hypertension (angiotensin infusion) and elastase injection. The diversity of protocols notwithstanding, we found that no model was consistent with the clinical definition of DCI in humans; meaning that no model confirmed the evidence of cerebral infarction with imaging plus neurological deficits.

Many publications came from a small number of teams. In fact, over half of publications came from only 14 teams. Different modalities were used for SAH induction with various origin of the blood, vascular territory involved, severity of SAH, method for the monitoring, and management of anesthesia. The inconsistency of SAH models may threaten the reproducibility of preclinical research. For instance, in models involving injection of blood in rodent brains, stereotactic coordinates guide the injection, but the diagnostic of SAH through imaging or necropsy was rarely performed. Similarly, endovascular perforation models did not control the quantity of blood released in cerebrospinal fluid and thus variability occurs at this level. Besides, different methods may result in different hemodynamic and homeostasis conditions thereby affecting cerebral perfusion differently. Another concern is the possible irrelevance of described models to humans. More than half (58%) of the here-described SAH models involve the posterior cerebral circulation. In contrast, in humans, SAH occurs in most cases (90%) in the anterior cerebral circulation. We found that arterial vasospasm was the most frequently assessed outcome (67%; *n* = 51). On the other hand, cerebral ischemia, and neurological status, which lie at the root of DCI definition, were the outcomes assessed in only 38 and 24% of publications respectively (Table 1). Although it is difficult to estimate accurately the rate of vasospasm in these preclinical studies given the lack of precise data on the incidence rate, vasospasm is probably overestimated. Indeed, vasospasm was evidenced in 87% of animals as compared to a two third proportion of vasospasm observed in humans, only half of whom are symptomatic (108). With respect to ischemia outcomes, only 4 publications assessed ischemia through imaging (43). For the first of them, the result is questionable given that the surgical procedure contributes to early ischemia. This result was not consistent with the experts' definition of DCI in humans since ischemia was assessed 1 h after SAH induction while the definition excludes early lesions (occurring in the first 48 h) and procedure-related infarctions. In most publications, ischemia was assessed through histology as illustrated on the 10 most cited studies included in our systematic review (Supplementary Table). Different histological techniques were used for ischemia assessment such as neuronal counting or detection of apoptosis. These modalities could be challenged because, as in vasospasm, the incidence of ischemia may be overestimated in comparison with epidemiological human data. In humans, however, DCI is routinely diagnosed through MRI or computed tomography scan. We trust it would be valuable to use imaging more often in preclinical settings since ischemia is well defined by imaging. We found that behavioral studies of animals were rarely performed, and no standardized protocol was used to diagnose neurological deficits after brain injury.

Regarding the literature on the same topic, a previous systematic review focused on *in vivo* models of vasospasm (4). Authors concluded that despite a great number of experimental SAH methods, no consistent models could be identified and recommended. In this review, 66 inductions method of SAH were identified. But there results were not only restricted to rodents. In contrast, in our systematic review we purposely restricted our search to only rodents given that this species are far the most used in biomedical research. Rodents have a well-characterized genome, with a high quantity, and quality of resources available for preclinical studies. Another review by Kamp et al. (109) focused on the mortality in mouse models analyzing DCI after SAH. They found that the mortality rate following aneurysmal SAH and DCI was significantly lower in mice than in humans. As in our review, the timing to assess mortality was not standardized in mouse models, potentially influencing the mortality rate. The authors concluded that further analyses would be required to establish a link between mortality and DCI models. This conclusion challenge DCI models themselves as well as their outcomes. In a last systematic review, Oka et al. screened SAH animal models and focused on DCI and neurological deficit. The authors equally found that preclinical models do not consistently lead to DCI (110). These conclusions further challenge DCI models as well as their outcomes.

In we look in the literature, several pharmacological treatments have been tested in the last few years to prevent or treat DCI. Unfortunately, most were either negative or led to only mild improvement in clinical outcome.

The only treatment which has shown an improvement in the functional outcome and which is currently recommended with a Level of Evidence grade A, by the American Heart Association/American Stroke Association for prophylactic treatment after SAH is Nimodipine (111). Nimodipine is a calcium channel blocker, which has largely been tested. A meta-analysis conducted in 2011 found a reduction of death or severe disability in patients treated with a prophylactic administration as compared to controls (112). Interestingly, cerebral infarctions were reduced in the treated group, but vasospasm was not significantly impacted.

Dorhout Mees et al. made a review about antiplatelet agents that have failed to show any beneficial effect on outcome (measure by death or handicap) and DCI (113). An antagonist of Endothelin-1, Clazosentan has shown some improvement in both vasospasm and DCI, demonstrated in the large meta-analysis over 1900 patients (114). However, the phase III clinical trial CONSCIOUS 2 reported that Clazosentan has no significant effect on mortality and vasospasm-related morbidity or functional outcome (115). Besides these two targets, several anti-oxidants agents have been tested at large scale, with no positive result on the outcome (116). Statins have also been studied in large phase III randomized and controlled trials, as a therapeutic strategy blocking different pathophysiological targets at the same time, but they failed to show any beneficial effect on outcome (117).

These results of clinical trials are interesting both to understand the pathophysiology of DCI and to design better experimental models. For instance, since it showed benefit on DCI in clinical trials, nimodipine could be used to demonstrate the clinical relevance of experimental models of SAH. In a clinically relevant model, nimodipine should have beneficial effects, whereas the other treatments presented above should not.

We hope these results will influence future SAH preclinical research. Our findings emphasize the need to standardize the method for DCI diagnosis through short and long-term behavioral motor, emotional and cognitive evaluations, histology, and/or imaging. Most of preclinical studies assessed solely intracranial vasospasm while it may not be a relevant outcome. Indeed, it has been shown in therapeutic clinical trials that pharmacological treatments can reduce the angiographic vasospasm without any effect on functional outcome or mortality (118). We believe that a comprehensive neurobehavioral assessment, mortality and imaging proof of ischemia should be the preferred outcomes in animal studies. This approach is consistent with recommendations for animal studies of ischemic stroke (119) or intracerebral hemorrhage (120). The methodological heterogeneity we observed in experimental SAH studies could also be found in pathologies such as stroke (121), intracerebral hemorrhage (120) or brain tumor (122). We trust that the assessment of ischemia through the association of neurological evaluation and imaging in experimental studies will enhance the quality of translational research.

Our review has some limitations. First, we excluded publications evaluating therapeutic agents. This may exclude a number of articles with SAH models, but we considered that the aim of these studies was not to describe new SAH models but to assess drugs' efficacy. Therefore, these studies were not considered. Moreover, most of these studies used SAH models previously described. Second, we selected only *in vivo* studies. This point can be questionable because one could consider *in vitro* studies more relevant to understand pathophysiological mechanisms and to test therapeutics. But such *in vitro* models cannot recapitulate all features of the DCI definition, in particular neurological outcome, so that preclinical animal models remain the only option.

Finally, in our meta-analysis we decided to focus on rodents and not include other animals. Our choice was justified a priori considering the prevalence of these species in biomedical research. These species offer several possibilities with genetically modified strains to focus on a therapeutic approach. Nevertheless, aware of this limit of our review, we also reviewed non-human primate models (but this was not a pre-planned analysis) (Table 2).

This review demonstrates first the high proportion of non-human primate models with blood injection either directly with perivascular clot placement or by injection into a cerebrospinal fluid cisterna (prechiasmatic or *cisterna magna*). These models have the disadvantage to shifting away from pathophysiological mechanisms involving aneurysm rupture and acute autologous arterial blood extravasation. However, non-human primate models have an unquestionable benefit for neurological examination to detect delayed neurological deficit. This is a crucial advantage over rodent models to make longitudinal examinations in the same animal and to relate more closely to the human disease, since the occurrence of neurological deficit is a diagnostic criterion of DCI.

Furthermore, as noted with rodent models, the study of vasospasm is largely overrepresented in non-human primate models (21 studies) compared to the study of DCI (3 studies) (Table 2). Thus, despite the possibility of more efficient clinical longitudinal follow-up in non-human primates, most of the studies did not take full advantage of the possibilities offered by these experimental models. Additionally, it is difficult to consider non-human primate models for exploratory research because of reproducibility, ethical issues, and cost. Non-human primates models could be envisaged for preclinical SAH research especially to monitor neurological status, in order to test therapeutic efficacy before clinical trial.

At the end of this review, we were able to highlight that no SAH model consistently lead to DCI rodents. In order to improve translational research, efforts should focus on developing clinical relevant models rather than continuing experimental studies with irrelevant models.

Moreover, we insist on future studies with an urgent need to develop SAH models focusing on the clinically relevant outcomes. Future studies should choose the appropriate experimental design study, in accordance with the existing data in DCI, while reflecting on the choice of species, SAH induction method and experimental study to answer the question from therapeutics and/or pathophysiological mechanistic.

Furthermore, researchers should respect principles of good laboratory practice with rigor and reproducibility as it is currently recommended (123), in order to standardize preclinical studies and results.

## Conclusion

We described 8 published preclinical SAH models for rats and mice. Some of them allow for the assessment of vasospasm and/or ischemia; however, none allows the assessment of DCI as the scientific community in humans defined it with association between neurological evaluation and brain imaging. We believe developing a consensual preclinical model matching the human description of DCI will help enhance translational research.

## Data Availability Statement

The original contributions presented in the study are included in the article/Supplementary Material, further inquiries can be directed to the corresponding author/s.

## Author Contributions

All authors listed have made a substantial, direct and intellectual contribution to the work, and approved it for publication.

## Funding

This work was supported by the French National Research Agency program PREDICT. SG was funded of Fondation pour la Recherche Medicale (FRM). Figures 1, 5 was created with BioRender.com.

## Conflict of Interest

The authors declare that the research was conducted in the absence of any commercial or financial relationships that could be construed as a potential conflict of interest.

## Publisher's Note

All claims expressed in this article are solely those of the authors and do not necessarily represent those of their affiliated organizations, or those of the publisher, the editors and the reviewers. Any product that may be evaluated in this article, or claim that may be made by its manufacturer, is not guaranteed or endorsed by the publisher.
