# Supplementary material for: Delayed Cerebral Ischemia After Subarachnoid Hemorrhage: Is There a Relevant Experimental Model? A Systematic Review of Preclinical Literature
Source: Front Cardiovasc Med. 2021 Nov 15;8:752769. doi: 10.3389/fcvm.2021.752769 (PMC8634441; doi:10.3389/fcvm.2021.752769)
Supplement: Supplementary file 1 [file Table_1.DOCX]

**Delayed cerebral ischemia after subarachnoid hemorrhage: is there a relevant experimental model? A systematic review of preclinical literature.**

**Suzanne Goursaud^1,2^, Sara Martinez de Lizarrondo^2^, François Grolleau^3^, Audrey Chagnot^2^, Véronique Agin^2^, Eric Maubert^2^, Carine Ali^2^, Maxime Gauberti^2^, Denis Vivien^2,4^, Clement Gakuba^2,3^**

Supplementary table:

Characteristics of the SAH models described in the ten most cited studies included in the systematic review *(NA: not available)***.**

| **Studies** | **Citations (number)** | **Animal species** | **Models** | **Blood volume** | **Vasospasm** | **Cerebral ischemia** | | |
| --- | --- | --- | --- | --- | --- | --- | --- | --- |
|  |  |  |  |  |  | **Imaging** | **Histology** | **Behavioral study** |
| *Bederson. 1995* | 360 | Rats | Endovascular perforation | Uncontrolled | NA | NA | NA | No focal neurological deficit |
| *Bederson.* 1998 | 245 | Rats | Endovascular perforation | Uncontrolled | Yes  (doppler and histology) | NA | NA | NA |
| *Delgado*. 1985 | 234 | Rats | Direct injection into the *cisterna magna* | 70 et 300 μL | Yes  (angiography) | NA | NA | NA |
| *Solomon.* 1985 | 175 | Rats | Direct injection into the *cisterna magna* | 300 μL | NA | NA | NA | NA |
| *Prunell.* 2003 | 175 | Rats | Direct injection into the *cisterna magna /* prechiasmatic cistern | 200 et 300 μL | NA | NA | Yes  (neuronal death in the hippocampus and the cortex) | Yes  (Hemiparesis observed in the SAH group) |
| *Jackowski.* 1990 | 154 | Rats | Direct injection into the *cisterna magna* | 300 μL | Yes  (histology) | NA | NA | NA |
| *Sugawara.* 2008 | 129 | Rats | Endovascular perforation | Uncontrolled | Yes  (histology) | NA | NA | Yes  (Severe to moderate neurological deficits observed in the SAH group) |
| *Veelken*. 1995 | 119 | Rats | Endovascular perforation | Uncontrolled | NA | NA | Yes  (neuronal loss in the hippocampus) | NA |
| *Gules*. 2002 | 112 | Rats | Direct injection into the *cisterna magna* | 300 μL | Yes  (histology) | NA | NA | No focal neurological deficit |
| *Doczi.* 1986 | 102 | Spontaneous hypertensive rats | Direct injection injection to the surface of the cortex | 200 μL | NA | NA | NA | NA |
